# Persistence and Potential Viable but Non-culturable State of Pathogenic Bacteria during Storage of Digestates from Agricultural Biogas Plants

**DOI:** 10.3389/fmicb.2016.01469

**Published:** 2016-09-14

**Authors:** Geraldine Maynaud, Anne-Marie Pourcher, Christine Ziebal, Anais Cuny, Céline Druilhe, Jean-Philippe Steyer, Nathalie Wéry

**Affiliations:** ^1^LBE, INRANarbonne, France; ^2^UR OPAALEIrstea, Rennes, France; ^3^Univ Bretagne LoireRennes, France

**Keywords:** anaerobic digestion, *Listeria monocytogenes*, *Salmonella*, *Campylobacter*, digestate, persistence, VBNC state

## Abstract

Despite the development of on-farm anaerobic digestion as a process for making profitable use of animal by-products, factors leading to the inactivation of pathogenic bacteria during storage of digestates remain poorly described. Here, a microcosm approach was used to evaluate the persistence of three pathogenic bacteria (*Salmonella enterica* Derby, *Campylobacter coli* and *Listeria monocytogenes*) in digestates from farms, stored for later land spreading. Nine samples, including raw digestates, liquid fractions of digestate and composted digestates, were inoculated with each pathogen and maintained for 40 days at 24°C. Concentrations of pathogens were monitored using culture and qPCR methods. The persistence of *L. monocytogenes*, detected up to 20 days after inoculation, was higher than that of *Salmonella* Derby, detected for 7–20 days, and of *C. coli* (not detected after 7 days). In some digestates, the concentration of the pathogens by qPCR assay was several orders of magnitude higher than the concentration of culturable cells, suggesting a potential loss of culturability and induction of Viable but Non-Culturable (VBNC) state. The potential VBNC state which was generally not observed in the same digestate for the three pathogens, occurred more frequently for *C. coli* and *L. monocytogenes* than for *Salmonella* Derby. Composting a digestate reduced the persistence of seeded *L. monocytogenes* but promoted the maintenance of *Salmonella* Derby. The effect of NH${}_{4}^{+}$/NH_3_ on the culturability of *C. coli* and *Salmonella* Derby was also shown. The loss of culturability may be the underlying mechanism for the regrowth of pathogens. We have also demonstrated the importance of using molecular tools to monitor pathogens in environmental samples since culture methods may underestimate cell concentration. Our results underline the importance of considering VBNC cells when evaluating the sanitary effect of an anaerobic digestion process and the persistence of pathogens during the storage of digestates and subsequent land spreading.

## Introduction

Anaerobic digestion (AD) is one option for managing organic waste originating from agriculture, industry or wastewater treatment. By converting wastes, AD generates both methane for use as energy and the residual digestate as a fertilizer which appears to be a very good candidate to replace inorganic fertilizers (Tambone et al., 2010). However, the use of digestate as fertilizer inevitably involves the issue of health risks associated with pathogenic microorganisms present in waste of fecal origin (such as manure, slurry or sludge): such pathogens may be spread along with the digestate on agricultural soils (Smith et al., 2004; Goberna et al., 2011; Johansen et al., 2013), with consequent contamination of the food chain. Among them, these pathogens number a variety of viruses, including swine hepatitis E virus, bacteria including *Salmonella* sp., *Listeria monocytogenes*, *Mycobacterium paratuberculosis*, thermotolerant *Campylobacter* and parasites including *Cryptosporidium parvum* and *Giardia* sp. (Avery et al., 2012). Among bacteria, the most studied pathogens belong to the genera *Salmonella*, *Campylobacter* and *Listeria*. According to the report of the European Food Safety Authority (EFSA, 2015), the numbers of human listeriosis further increase since 2008 (increase of 16% from 2013 to 2014). Although the number of cases is relatively low, the death rate is higher than other foodborne diseases. Campylobacteriosis was the most commonly reported zoonosis with an increase in confirmed human cases in the European Union since 2008 (increase of 10% from 2013 to 2014). Salmonellosis cases increase for the first time since 2008. From 2008 to 2014, salmonellosis decreased by 44%, mainly due to the successful *Salmonella* control programs.

The behavior of pathogens during AD has been studied by several authors (Smith et al., 2005; Wagner et al., 2009; Avery et al., 2014; Fu et al., 2015). Temperature appears to be the main factor affecting pathogen viability during AD (Wagner et al., 2009). Thermophilic AD reduces the quantity of pathogens more efficiently than mesophilic AD which only reduces the concentration of pathogens by 1–2 log units (Avery et al., 2012). Due to their insufficient elimination during the AD process (Pietronave et al., 2004), pathogens may be still present in low concentrations in the digestate. Many confined-livestock farms store their waste or final products for several months prior to use as fertilizer (Himathongkham et al., 1999). However, very little information about the viability of pathogens during the storage of digestates has been reported. Furthermore, some studies have shown potential regrowth of coliforms and pathogens during the storage of compost (Sidhu et al., 2001; Pietronave et al., 2004), biowaste (Gibbs et al., 1995; Bagge et al., 2005) or digestate (Fu et al., 2015; Orzi et al., 2015). Sidhu et al. (2001) did not observe a decline of *Salmonella* sp. when the compost was stored. These authors highlighted a strong negative correlation between the *Salmonella* inactivation rate and the maturity of the compost. They suggested that long-term storage could facilitate the regrowth of pathogens due to a decline in the antagonist effect of indigenous microorganisms.

Another issue concerns the loss of culturability of bacteria during waste treatment. In fact, several studies have demonstrated that pathogens can enter into a Viable but Non-Culturable (VBNC) state in biosolids or animal by-products after various treatments, including the AD process (Higgins et al., 2007; Qi et al., 2008; Viau and Peccia, 2009; Fu et al., 2014; Desneux et al., 2016). Fu et al. (2015) demonstrated the induction of a VBNC state of *Salmonella* sp. and *Shigella* sp. during the thermophilic AD of biosolids but followed during cake storage with a subsequent regaining of culturability. Exposure to various stresses can induce the VBNC state and VBNC cells may be reactivated back to a culturable state under suitable stimuli (Li et al., 2014). The presence of VBNC bacteria in the environment constitutes a health risk for humans since the cells may retain their potential to infect (Besnard et al., 2001).

In order to improve risk assessment of animal by-products when re-used in agriculture, we investigated the persistence during the storage of digestates of three pathogenic bacteria found in animal feces: *Salmonella* Derby (Huong et al., 2014), *Campylobacter coli* (Schweitzer et al., 2011; Egger et al., 2012) and *Listeria monocytogenes* (Desneux et al., 2016). The persistence of pathogens in nine digestates from farms practicing land spreading (raw digestates, liquid fractions of digestate after separation and dried or composted solid fractions of digestate) was studied via microcosm assays lasting 40 days. More especially, the goals were to: (i) test different digestates for inactivation of pathogenic bacteria and to identify the characteristics of digestates affecting their persistence; and (ii) study the occurrence of a VBNC state in digestates using both the quantitative PCR (qPCR) assay and culture methods.

## Materials and Methods

### Sampling of Digestates

Nine digestates (named Dig1–9) originating from wet mesophilic AD were sampled from seven agricultural sites in France, gathering different types of digestates that can be spread on soil: three raw digestates (Dig1–3), three liquid fractions of digestate (Dig4–6) obtained after digestate separation, one composted and dried fraction of digestate (Dig7) and two composted solid fractions of digestate (Dig8–9). Digestate designation and their origin are presented in **Table 1**. To evaluate the variability due to sampling and the analytical variability, two independent samples were collected for digestates Dig2 and Dig4, on the same day following the same sampling protocol (named R1 and R2). All the collected samples (about 50 kg each) were homogenized for further analyses.

**Table 1:** Designation and origin of digestates.

Digestate	Nature of sample	Main digester feedstock	Process of digestion			Phase separation of digestate	Quantity of waste digested per year (T/year)
			Total retention time^2^ (days)	Temperature (°C)	Post-digestion		
Dig1	Raw digestate	Pig slurry, fat, green waste	80	42	Yes	No	6000


Dig2	Raw digestate	Cattle slurry and manure, vegetable waste, lactose	20–30	37	No	No	5500


Dig3	Raw digestate	Cattle manure and slurry, filtration sediment residues, lactose permeate^1^	15–16	38	Yes	No	12000


Dig4	Liquid fraction	Pig slurry, food processing waste, maize waste	50–60	38	No	Drum filter	12500


Dig7	Composted and dried solid fraction	Pig slurry, biowaste, WWTP^3^ sludge, fat	44	38	Yes	Centrifugation	12000


Dig5 Dig8	Liquid fraction (Dig5) and compost of solid fraction (Dig8)	Manure, green waste	30–32	40	No	Centrifugation	5000


Dig6 Dig9	Liquid fraction (Dig6) and compost of solid fraction (Dig9)	Cattle manure and slurry, poultry manure, green waste	60	41	No	Screw press	6000

^1^*Wastes are pre-treated for 2 days by hydrolysis at 42°C, ^2^including the time of the post-digestion when done, ^3^wastewater treatment plant*.

### Physical and Chemical Characterization of Digestates

Representative aliquots of digestate (10–50 g for composted digestates and 10–50 mL for raw digestates and liquid fractions) were used in carrying out all the analytical tests. For each samples, the moisture content was determined by drying the sample at 105°C for 48 h. The volatile matter (VM) was measured by calcination of this dried sample at 550°C for 3 h. The pH was measured in accordance with the AFNOR standard NF EN 15933: for solid digestates (<60% moisture content, Dig7–9), in the aqueous extract obtained after adding distilled water (1:5, w/v), or directly for liquid digestates (>88% moisture content, Dig1–6). Total organic carbon (TOC) was analyzed using a liquid-phase carbon analyzer (TOC-V CHS/CSN, Shimadzu Corporation). Total nitrogen was determined using the Total Kjeldahl Nitrogen (TKN) method (Rodier, 1975). Ammonia (NH${}_{4}^{+}$) concentration was determined by high-performance liquid chromatography (HPLC) using the manufacturer’s protocol (ICS-3000 Ion Chromatography System). Total Phosphorus (P_2_O_5_) content was measured by colorimetric determination, in accordance with Fiske and Subbarow (1925). Total Potassium (K_2_O) content was measured in accordance with the AFNOR standard NF EN ISO 11885. TOC, NH${}_{4}^{+}$, TKN, P_2_O_5_ were analyzed in fresh samples whereas K_2_O was analyzed in dried samples. All the analyses were performed in triplicate and showed variability below 10%. All analyses were expressed as g of raw matter.

### Bacterial Strains and Culture Conditions

The bacterial strains used were *L. monocytogenes* strain CIP110870 and *Salmonella enterica* subsp. *enterica* serovar Derby strain CIP110872 (*Salmonella* Derby), both originating from pig manure, and a gentamicin-resistant *Campylobacter coli* strain UB14-031/1 of clinical origin (provided by the French National Reference Center for *Campylobacter* and *Helicobacter*, Bordeaux). To facilitate *L. monocytogenes* and *Salmonella* enumeration on plate agar, two rifampicin-resistant (Rifr) mutants of *L. monocytogenes* and *Salmonella* Derby were generated from the two selected strains, as described in Lung et al. (2001). The Rifr strains were cultivated at 37°C in nutritive broth (ThermoFisher Scientific, France) supplemented with 3 g L^-1^ of glucose. *C. coli* was cultivated under microaerobic conditions at 42°C in nutrient Broth No.2 (ThermoFisher Scientific), supplemented with 5% of horse blood (v:v) and *Campylobacter* growth supplements (ThermoFisher Scientific). The plates were placed in a microaerobic environment using CampyGen (ThermoFisher Scientific) which produces 6–15% O_2_ and 3–9% CO_2_ (Macé et al., 2015). The broths inoculated with *L. monocytogenes*, *Salmonella* Derby and *C. coli* were incubated for, respectively 18, 17, and 28 h, to reach the beginning of the stationary phase.

### Microcosm Assay Inoculated with Live Cells

Each 120 mL of the pathogen culture suspension in its stationary phase was centrifuged at 5000 *g* for 10 min. The pellet was washed in 30 mL of 0.8% of sterilized NaCl solution. Following a second step of centrifugation, the pellet was resuspended in 10–12 mL of NaCl solution prior to inoculation in the digestates. Microcosms, established in 500 mL flasks with plastic screw cap lids, contained 125 mL or 100 g of digestate. Each microcosm was inoculated with 1 mL of *L. monocytogenes*, *Salmonella* Derby or *C. coli* culture suspension to reach an initial concentration of 10^6^–10^7^ CFU mL^-1^ or g^-1^. The inoculated flasks were stored in the dark at 24°C for 40 days. There were two replicates per digestate. According to the nature of the digestate (liquid or solid), the microcosms were prepared in accordance with the two following protocols: for liquid digestates (Dig1–6), a volume of 125 mL was transferred into a 500 mL flask, and for solid digestates (Dig7–9), 100 g were transferred into five 500 mL-flasks and at each sampling one flask was sacrificed by adding 300 mL of peptone water. At each sampling time, aliquots for cultural (1 mL) and qPCR (500 μL) quantification were collected immediately after inoculation of the strains (t0) and then after 3, 7, 20, and 40 days of incubation. Aliquots for qPCR were stored at -20°C before analysis.

### Control Assay Inoculated with Killed Cells

A second series of microcosms was prepared in the same way with the bacterial suspensions in a NaCl solution after the cells were lysed by ultrasound for 8 min at 360 W and then heated to 85°C and maintained here for 10 min. The absence of culturable cells was checked by inoculation onto a culture medium as described above. Before inoculation in the flasks, the suspensions of killed cells were stored at -20°C. Each microcosm was performed in duplicate. The microcosms inoculated with killed cells were prepared and incubated in the same way as for the previous microcosms. The concentration of DNA of each pathogen was measured by qPCR at the beginning of the experiment (t0) and thereafter 1, 2, 5, 7, 20, and 40 days of incubation.

### Enumeration of Pathogens and Endogenous *Escherichia coli* by Culture Method

Serial 10-fold dilutions were prepared in peptone water (ThermoFisher Scientific). A volume of 0.1 mL of each dilution was surface plated on the specific medium. When the level of bacteria was expected to be low, 0.1 mL of the digestate was surface plated on one agar plate. *E. coli* was enumerated on Tryptone Bile X-Glucuronide (TBX, ThermoFisher Scientific) agar plates. The plates were incubated at 44°C for 24 h. After incubation, blue colonies (glucuronidase positives) were counted. *L. monocytogenes* and *Salmonella* Derby strains were enumerated on Palcam agar (ThermoFisher Scientific) and on XLD agar (ThermoFisher Scientific), respectively, supplemented with 50 mg L^-1^ of cycloheximide and 100 mg L^-1^ of rifampicin (Sigma Aldrich, France). The plates were then incubated 48 h at 37°C. For *C. coli*, the cells were enumerated on Karmali-Agar (ThermoFisher Scientific), supplemented with Karmali selective supplement and 32 mg L^-1^ of gentamicin, then incubated for 72 h at 42°C under microaerobic conditions using CampyGen (ThermoFisher Scientific). Results are expressed as CFU mL^-1^ or g^-1^. The limit of detection was 25 CFU mL^-1^ or g^-1^.

### Quantification of Pathogens by qPCR

DNA extraction was performed on 500 μL of digestate using the FastDNA SPIN Kit for soil, in accordance with the manufacturer’s protocol (MP Bio, Solon, OH, USA). The quantity and quality of the DNA were checked by spectrophotometry (Infinite NanoQuant M200, Tecan, Austria). All the DNA was stored at -20°C until further use.

Reactions took place using the concentrations of oligomers and the PCR programs described in **Table 2**. Reactions were prepared in 25 μL, using 96-well optical reaction plates (Applied Biosystems, Foster City, CA, USA). The following components were added: 12.5 μL of 2X SsoAdvanced universal supermix (Bio-Rad, Hercules, CA, USA), 5 μL of diluted DNA extracts, the forward primer, reverse primer and the TaqMan^TM^ probe labeled with FAM as a reporter and TAMRA as a quencher. Amplification reactions were run on the CFX96 Real-Time system (Bio-Rad). All tests were performed in triplicate. Data analysis was carried out with the Bio-Rad CFX Manager software, version 3:0. The cycle threshold (Ct), which corresponds to the cycle number at which the reaction became exponential, was then compared to a standard curve and used to calculate the number of target per PCR.

**Table 2:** Conditions for qPCR analysis.

Target bacteria and gene	Amplicon (bp)	Concentration of primers (nM)			PCR programs^∗^	Reference
		Forward	Reverse	Probe		
*Salmonella* Derby *inv*A	141	150	150	100	15 s at 95°C, 1 min at 58°C	Hoorfar et al., 2000
*Listeria monocytogenes hly*A	133	200	200	50	15 s at 95°C, 1 min at 58°C	Nogva et al., 2000
*Campylobacter coli gly*A	147	150	150	100	15 s at 95°C, 1 min at 54°C	Leblanc-Maridor et al., 2011

*^∗^Repeated for 40 cycles, after an initial cycle of 2 min at 95°C*.

One standard curve was generated for each assay, using plasmid containing the target gene (MWG Operon Eurofin, Ebersberg, Germany). The standard curves designed by the Bio-Rad CFX Manager software were then generated by the amplification of serial 10-fold dilutions of the plasmid solutions in sterilized water. Slopes of standard curves were between -3.1 and -3.5, corresponding to an efficiency of the PCR reaction between 90 and 110%. The regression coefficient values (*R*^2^) were always above 0.95.

No background signal for any of the qPCR systems was detected in the control which was obtained by replacing DNA by sterilized water. To validate the absence of PCR inhibition, internal control tests were performed. One microliter of 20X PrimePCR positive-control probe assay (labeled with HEX as a reporter and lowa Black as a quencher) (Bio-Rad) was added to wells containing 5 μL of DNA extracts as well as to wells containing DNA free water. All were done in triplicate. The Ct obtained with and without DNA extracts were compared and a different Ct (±0.5) indicated an inhibition of the PCR reaction.

The level of background signal and the efficiency of the PCR were checked for each assay. The PCR results were then converted to target gene copy numbers per g of raw matter. Average values and standard deviation were obtained using at least three values. The detection limit was determined for each sample since the quantity of sample used for DNA extraction varied from one sample to another. The detection limits were between 1.9 × 10^3^ and 1.0 × 10^4^ copies g^-1^.

### Analysis of Pathogen Quantifications by qPCR and Culture Method

Quantification of DNA or enumeration of CFU recovered from each sampling day and for each pathogen was normalized by dividing the mean values by the mean obtained at day 0 (C/C0). We performed a Log transformation of the ratio Log(C/C0) and plotted these values against the sampling day for each pathogen and each digestate. Two parameters describing the persistence of pathogen were calculated: the decimal reduction at 7 days and the inactivation rate (d^-1^) corresponding to the slope of the inactivation curve and expressed in Log.

The number of culturable cells was estimated by plate count and the number of total cells was estimated by qPCR. Given that the target genes amplified by qPCR (*hly*A, *inv*A and *gly*A) are present as a single copy in the genome for the three pathogenic bacteria (**Table 2**), results can be expressed as a genome equivalent. The difference between the concentration of total cells measured by qPCR (C_qPCR_) and the concentration of culturable cells (C_culture_)_,_ expressed in Log, (Log C_qPCR_ – Log C_culture_) was calculated at 7 days for *C. coli* and *Salmonella* Derby and at 20 days for *L. monocytogenes* (before the initial lag phase), with both C_qPCR_ and C_culture_, expressed as genome equivalent mL^-1^ or g^-1^. The presence of potential VBNC cells was assumed when (Log C_qPCR_ – Log C_culture_) was significant, taking into account the coefficient of variation (CV) of each quantification method. Moreover, killed cells control was used to confirm that the qPCR signal of microcosms inoculated with killed cells decreased at a higher rate than the qPCR signal of microcosms inoculated with live cells. Potential VBNC state was validated if the qPCR signal from killed cells was significantly below the qPCR signal of live cells.

### Statistical Analysis

The mean, standard deviation and CV of physical and chemical analysis and pathogen quantification in Log at 7 days were calculated for the two digestates sampled twice (Dig2 and Dig4). A Pearson correlation matrix was performed in order to test for correlations between the physical and chemical characteristics of a digestate (pH, moisture content, VM, TOC, NTK, NH${}_{4}^{+}$, K_2_O and P_2_O_5_) and the persistence of pathogens (decimal reduction and inactivation rate). Statistical analyses were carried out using the Rcmdr package of R-Software (R Foundation for Statistical Computing, Vienna, Austria).

## Results

### Comparison of the Persistence of the Three Pathogenic Bacteria in Digestate Microcosms

**FIGURE 1:**
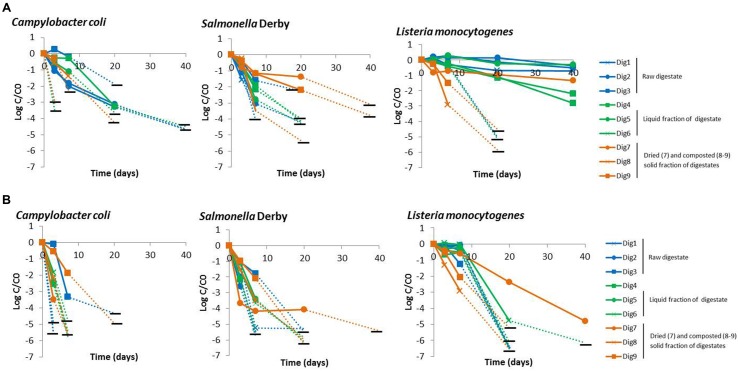
**Persistence of *C. coli*, *Salmonella* Derby and *L. monocytogenes* measured by qPCR assay **(A)** and the culture method (B)**. Only one microcosm duplicate is presented. Blue, green and orange curves represent respectively, raw digestates, liquid fraction of digestate and composted solid fractions of digestate. Horizontal black lines represent the detection limits. The last point connected by a dotted line represents the first point below the detection limit.

The nine digestates spiked with cells of *L. monocytogenes*, *Salmonella* Derby and *C. coli* were monitored for 40 days by both the culture method and qPCR assay. The variability of microbial, physical and chemical analyses was tested on two independent samples R1 and R2, collected from different digestors (Dig2 and Dig4). Taking into account the two samples and the two replicates per sample, the CV was below 12% (Supplementary Table S1) indicating data’s good reproducibility. Furthermore, the inactivation curves show good reproducibility between duplicates (Supplementary Figure S1). The behavior of each pathogen in each digestate is presented in **Figure 1**. Generally, concentrations declined from t0 exponentially. In some cases, the inactivation curve appears to have two parts, the first with a lag phase during which concentrations remained stable until day 3 or 7, followed by a second phase of sharp decline. Two parameters were chosen to characterize all inactivation curves: decimal reduction at 7 days and the inactivation rate (Supplementary Table S2). This profile with the lag phase was observed for samples Dig3–4 of *C. coli*, and for half the samples of *L. monocytogenes*. For these digestates, the inactivation rate was calculated after the lag phase.

The three pathogens survived differently in the digestates (**Figure 1**). Overall, *L. monocytogenes* showed the highest persistence. It was detected by qPCR in all digestates after 20 days and was still present after 40 days in 5 of the 9 digestates (Dig1–2, Dig4–5, and Dig7). Furthermore, the concentration measured by qPCR in 3 of the 9 digestates (Dig1–2 and Dig5) did not decrease over time. A high persistence during the first days of storage was also observed using the culture method with no decay at 7 days for five digestates. In contrast, *C. coli* was not detected by the culture method in most digestates (6 of the 9 digestates) after 7 days and the detection limit was also rapidly reached for quantification using qPCR. *Salmonella* Derby showed an intermediate persistence. It was quantified during 7–20 days and was still detected by qPCR up to 7 days in most of the digestates (7 of the 9 digestates).

### Potential Induction of VBNC State

**FIGURE 2:**
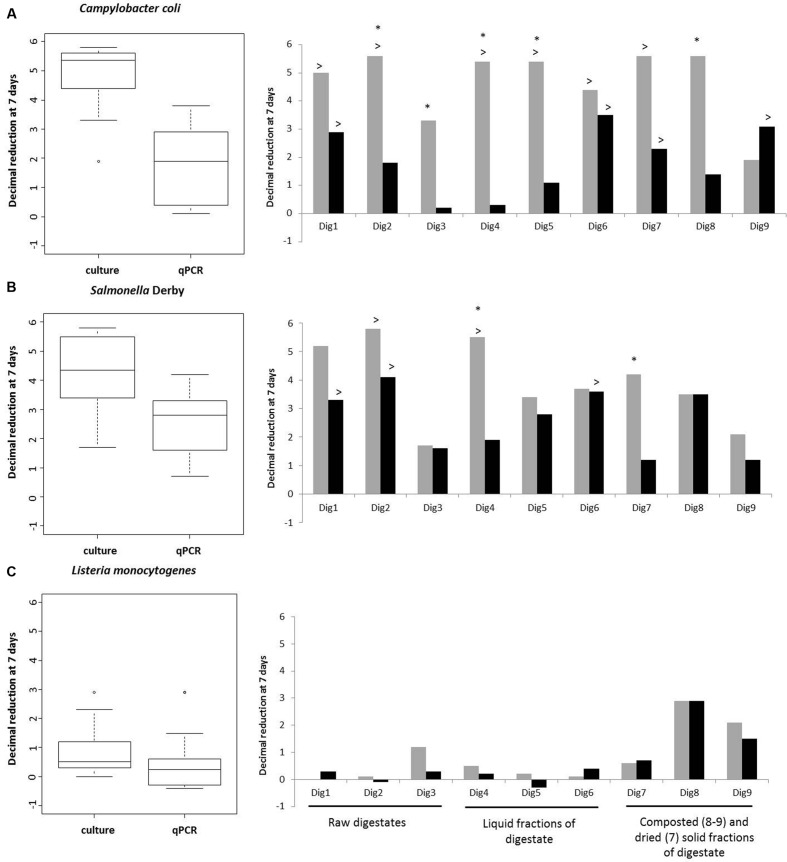
**Distribution of the decimal reductions among digestates in left panel and decimal reduction for each digestate in right panel, for *C. coli***(A)**, *Salmonella* Derby **(B)** and *L. monocytogenes***(C)**, measured by qPCR assay (black bars) and the culture method (gray bars)**. The symbol > significates that the decimal reduction is calculated using the detection limit. The symbol ^∗^ shows digestates with a potential induced VBNC state.

To test whether the culturability was lost faster than viability and if the induction of VBNC state was occurring, decimal reduction by qPCR and the culture method were compared for each pathogen after 7 days of incubation (**Figure 2**). As shown in the boxplots of **Figure 2**, high variability was observed, both by culture or qPCR, for *C. coli* with values ranging between 1.7 and 5.8 for culture and between 0.1 and 3.5 for qPCR. For *Salmonella* Derby, concentration varied from 1.7 to 5.8 for culture and from 0.7 to 4.1 for qPCR. The variability was lower for *L. monocytogenes*: from 0 to 3.3 for culture and from -0.3 to 2 for qPCR. Different behavior was observed depending on the digestate and the pathogen.

For *C. coli*, very different inactivation curves were obtained depending on the digestate. The detection limit of both methods was reached in three digestates (Dig1 and Dig6–7). A reduction of more than 5 Log of culturable cells associated to a relatively low reduction of gene copies (0.1–3.8 Log) was observed in four digestates (Dig2, Dig4–5 and Dig8), suggesting a potential induction of a VBNC state. In two digestates (Dig3 and Dig9), the culturable cells were less affected by the 7 days of storage in so far as their concentrations decreased by only 1.9 (for Dig9) to 3.3 Log (for Dig3). It is noteworthy that the decimal reduction measured by qPCR was 0.2 in Dig3 but reached more than 3 Log in Dig9. These results suggested a drastic effect of the digestate on *C. coli* persistence.

The behavior of *Salmonella* Derby also differed from one digestate to another. The highest decimal reductions of culturable cells (more than 5 Log) were observed in digestates Dig1-2 and Dig4. In some digestates, the decimal reduction observed by both techniques was similar (around 2 Log for Dig3 and around 3 Log for Dig5–6 and Dig8). In others, the difference between the culture and qPCR methods could not be calculated because the concentration was below the detection limit (for Dig1-2). For Dig4 and Dig9, the difference was above 1.9 Log. It is noteworthy that this difference increased with time, and at 20 days, significant differences were also observed for Dig9 (**Figure 1**).

**FIGURE 3:**
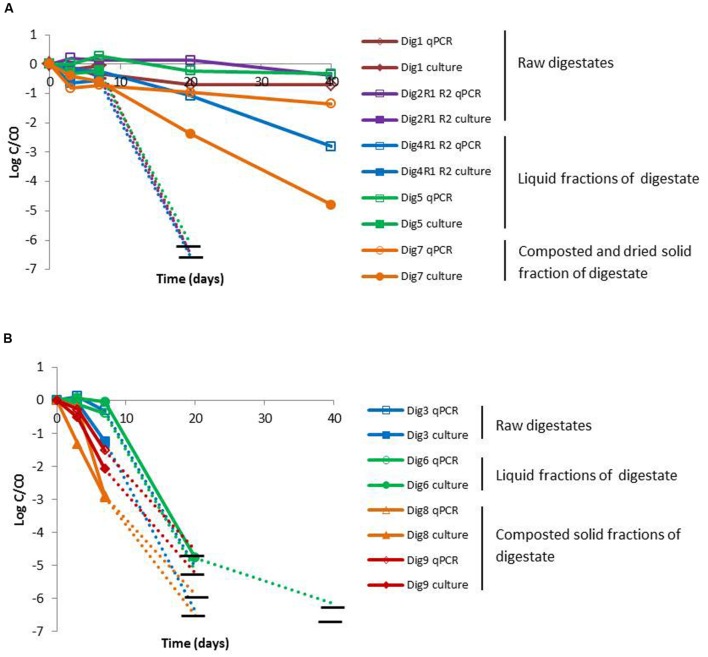
**Persistence of *L. monocytogenes* according to the difference between the quantification of cells by qPCR assay and the culture method: significant difference between the two methods ( > 1 log) after 20 days of storage **(A)** and no difference **(B)****. Empty and full symbols represent quantification by qPCR assay and the culture method, respectively. Horizontal black lines represent the detection limit. The last point connected by a dotted line represents the first point below the detection limit.

For *L. monocytogenes*, the decimal reduction after 7 days was less than 0.5 Log in 7 of the 9 digestates. However, two composted digestates (Dig8–9) led to 2 decimal reductions of the culturable cells and 1 (for Dig9) to 2 (Dig8) decimal reductions of gene copies. After the lag phase, the concentrations of culturable cells of *L. monocytogenes* decreased by at least 5 Log, regardless of the digestate (**Figure 3**). Two types of persistence behaviors as measured by qPCR were observed. In the first type of behavior, the qPCR signal either remained stable (Dig1–2 and Dig5) or decreased slightly (Dig4 and Dig7) (**Figure 3A**), suggesting that *L. monocytogenes* remained viable, with no drastic loss of viable cells, over 40 days but lost its culturability after 7 days, possibly entering into a VBNC state. In contrast, in four digestates (Dig3, Dig6, Dig8–9) the inactivation curves given by qPCR and the culture method were close to each other throughout the experiment (**Figure 3B**).

Overall, a potential VBNC state was observed for the three pathogenic bacteria but it was not systematic. The loss of culturability of *C. coli* and *Salmonella* Derby, which appeared in the first days of incubation, appeared later for *L. monocytogenes*. Although the loss of culturability depended on the digestates, the type of digestate (raw, liquid fraction or composted) alone cannot explained the loss of culturability. According to the coefficient of variation (CV) of the culture and PCR methods, the difference in decimal reduction above 1 Log was considered as indicator of a potential VBNC state. Potential loss of culturability was thus observed in five digestates (Dig2–5 and Dig8) in the case of *C. coli*, in three digestates (Dig4, Dig7, and Dig9) for *Salmonella* Derby and in five digestates (Dig1–2, Dig4–5, and Dig7) for *L. monocytogenes*.

To check if the higher values of qPCR could be due to the presence of no-degraded DNA from non-viable cells, the decrease of the qPCR signal obtained from killed cells of the three pathogens spiked in the nine digestates was determined and compared to the decrease obtained with live cells. **Table 3** presents the concentrations of pathogens measured in microcosms spiked with live cells by using qPCR assays and the culture method and in microcosms spiked with killed cells by using qPCR. A potential VBNC state was validated by a qPCR signal from killed cells significantly lower than the qPCR signal of live cells. The level of significance was given by the CV of each qPCR signal: 0.5 Log for *C. coli*, 1 Log for *Salmonella* Derby and 0.2 Log for *L. monocytogenes* (Supplementary Table S1). A potential VBNC state was validated for most of the digestates inoculated with killed cells. The validation could not be made for the composted and dried sample Dig7 which presented a very low decrease in the qPCR signal from killed cells that could be explained by the very low moisture content which prevents microbial and DNAse activity. Apart from the two microcosms performed with this digestate, a potential VBNC state was demonstrated for all the remaining 11 microcosms spiked with killed cells: 5 of the 9 digestates for *C. coli*, 2 for *Salmonella* Derby and 4 for *L. monocytogenes*.

**Table 3:** Validation of the degradation of DNA from non-viable cells for digestates presenting significant differences between qPCR and culture values (>1 Log) for *C. coli* **(A)**, *Salmonella* Derby **(B)** and *L. monocytogenes* **(C)**.

Digestate	C_culture_	C_qPCR_	Killed cells control C_qPCR_	C_qPCR_-C_culture_
**(A)**
Dig2	<DL	6.1	<DL	4.7^∗^
Dig3	2.4	7.5	<DL	5.1
Dig4	<DL	7.5	<DL	6.1^∗^
Dig5	4.4	6.4	<DL	2.0
Dig8	4.8	7.7	<DL	2.9
**(B)**
Dig4	<DL	6.2	5.2	4.8^∗^
Dig7	2.6	6.6	7.9	NV
Dig9	5.5	7.3	5.2	1.8^∗^
**(C)**
Dig1	<DL	8	<DL	6.6^∗^
Dig2	<DL	8.8	8.2	7.4^∗^
Dig4	<DL	7.7	5.8	6.3^∗^
Dig5	<DL	8.3	5.9	6.9^∗^
Dig7	5.1	7.8	8.1	NV

*Concentrations (Log) are given at 7 days for *C. coli* and *Salmonella* Derby and at 20 days for *L. monocytogenes*. The level of significance to compare the two qPCR signals (live cells vs. killed cells) was given by CV of each qPCR system (see Supplementary Table S1): 0.5 Log for *C. coli*, 1 Log for *Salmonella* Derby and 0.2 Log for *L. monocytogenes.* DL, detection limit; NV, not validated (killed cells control above qPCR signal). ^∗^The calculation was performed using the value of the detection limit for culture methods (25 CFU mL^-*1*^or g^-*1*^)*.

### Correlation between Physical and Chemical Characteristics of Digestates and Persistence of Pathogens and the Potential Induction of VBNC State

**Table 4** summarizes the physical and chemical characteristics of the digestates. The pH of all the digestates was alkaline, in a range of 7.3–8.8. The moisture content ranged between 25.4 and 59.1% for composted or dried solid fractions of the digestate (Dig7–9) and between 88.6 and 95.8% for raw digestates and liquid fractions of digestate (Dig1–6). These ranges of pH and moisture content were theoretically favorable to microbial activity, except for Dig7 which presented the lowest moisture content (25.4%). In addition to the moisture content, composted and dried digestates presented higher concentrations of VM (19.2–45.7%), TOC (94.8–235.8 gC kg^-1^), TKN (8.4–32.5 gN kg^-1^), K_2_O (7.3–12.8 g kg^-1^) and P_2_O_5_ (16.2–88.8 g kg^-1^) than liquid fractions and raw digestates. Finally, the NH${}_{4}^{+}$ content was between 0.1 and 3.3 g kg^-1^ for all digestates, except for Dig4 which contained a particularly high concentration of NH${}_{4}^{+}$ (7.9 g kg^-1^).

**Table 4:** Physical and chemical characteristics of digestates.

Digestate	pH	Moisture content (%)	VM (%)	TOC (gC kg^-1^)	TKN (gN kg^-1^)	NH${}_{4}^{+}$ (g kg^-1^)	K_2_O (g kg^-1^)	P_2_O_5_ (g kg^-1^)
Dig1	8.1	95.8	2.9	17.5	4.7	3.3	2.7	1.5


Dig2	8.0	91.2	3.4	19.6	4.2	3.2	8.0	3.3


Dig3	7.7	89.8	5.2	24.8	1.6	1.1	3.4	2.0


Dig4	8.3	93.8	3.5	20.4	10.2	7.9	2.9	5.8


Dig5	8.8	97.4	1.6	8.7	2.8	2.9	2.9	0.9


Dig6	8.0	88.6	5.0	33.5	3.7	2.4	6.3	1.9


Dig7	7.3	25.4	45.7	235.8	32.5	3.1	10.8	88.8


Dig8	7.8	56.1	22.7	116.1	12.5	1.9	7.3	45.2


Dig9	7.0	59.1	19.2	94.8	8.4	0.1	12.8	16.2

*VM, volatile matter; TOC, total organic carbon; TKN, total Kjedahl nitrogen*.

Pearson’s *r* correlations were calculated between physical and chemical parameters and the persistence of pathogens (inactivation rate and decimal reduction at 7 days) (**Table 5**). The persistence of the three pathogens did not correlate to the same parameters. The decimal reduction of *C. coli* was only significantly correlated to NH${}_{4}^{+}$ content (positively for culturable cells and negatively for gene copies), suggesting that NH${}_{4}^{+}$ /NH_3_ could have a negative effect on culturability but not on the viability of the bacteria. The decimal reduction of culturable cells of *Salmonella* Derby was also correlated only to NH${}_{4}^{+}$ content. However, significant correlation was found between the inactivation rate of the gene copies of this pathogen and several parameters: positively correlated to the moisture content and negatively to the nutrient content (TOC, TKN, P_2_O_5_) and VM. Composted digestates were characterized by low moisture content, high nutrient contents and VM. The correlation observed for *Salmonella* Derby thus indicated a higher persistence in composted digestates than in raw digestates and in liquid fractions. The decimal reduction at 7 days or the inactivation rate of *L. monocytogenes* measured by both methods was positively correlated to the nutrient content and VM and negatively correlated to the moisture content, thus indicating a lower persistence of this bacterium in composted digestates than in raw digestates and the liquid fraction of digestates.

**Table 5:** Pearson’s *r* correlation analysis between physical and chemical characteristics of digestates and pathogen inactivation rate (IR) and decimal reduction at 7 days (DR) calculated by both qPCR or the culture method and the difference in concentrations between qPCR assay and the culture method (data from **Table 3**) for *C. coli* **(A)**, *Salmonella* Derby **(B)** and *L. monocytogenes* **(C)**.

		TOC	K_2_O	TKN	NH${}_{4}^{+}$	P_2_O_5_	pH	Moisture %	VM %
(A) *Campylobacter coli*	Culture DR	-0.1	-0.4	0.2	**0.6^∗∗^**	0.1	0.2	0.2	-0.1
	Culture IR	-0.3	-0.1	-0.2	0.1	-0.2	0.1	0.3	-0.3
	qPCR DR	0.2	0.4	0.0	**-0.5^∗^**	0.1	-0.3	-0.2	0.2
	qPCR IR	0.1	0.2	-0.1	-0.4	-0.1	-0.4	-0.1	0.1
	Log C_qPCR_-Log C_culture_	-0.4	-0.4	-0.2	**0.5^∗^**	-0.2	0.3	0.3	-0.4

(B) *Salmonella* Derby	Culture DR	-0.2	-0.2	0.1	**0.7^∗∗^**	-0.1	0.2	0.2	-0.2
	Culture IR	-0.1	-0.1	0.1	**0.6^∗∗^**	0.0	0.1	0.2	-0.1
	qPCR DR	-0.4	-0.2	-0.4	0.0	-0.3	0.0	0.4	**-0.4^∗^**
	qPCR IR	**-0.6^∗∗^**	-0.3	**-0.6^∗∗^**	0.0	**-0.5^∗^**	0.0	**0.6^∗∗^**	**-0.6^∗∗^**

(C) *Listeria monocytognes*^1^	Culture DR	0.4	**0.4^∗^**	0.2	**-0.4^∗^**	0.3	-0.1	**-0.5^∗^**	0.4
	qPCR DR	**0.5^∗^**	0.4	0.3	-0.3	**0.5^∗^**	-0.2	0.3	**0.5^∗^**
	qPCR IR	0.1	0.1	-0.2	**-0.4^∗^**	0.0	**-0.5^∗^**	-0.2	0.1
	Log C_qPCR_-Log C_culture_	**-0.6^∗∗^**	**-0.5^∗^**	-0.4	**0.6^∗∗^**	**-0.5^∗^**	**0.7^∗∗∗^**	**0.7^∗∗∗^**	**-0.6^∗∗^**

*Significant correlations (^∗∗∗^*p-values* < 0.001, ^∗∗^*p-values* < 0.01, and ^∗^*p-values* < 0.05) are in bold. ^1^The inactivation rate measured by the culture method was not used in correlation analysis because it was calculated mainly using the detection limits. TOC, total organic carbon; TKN, Total Kjedahl nitrogen; VM, volatile matter*.

For *C. coli* and *L. monocytogenes*, correlations were also investigated between the potential VBNC concentration (estimated by the difference of concentrations between qPCR and culture), and the physical and chemical characteristics of the digestates (**Table 5**). This was not investigated for *Salmonella* Derby since potential induction of a VBNC state was shown for only two digestates. The difference in *L. monocytogenes* concentration between the two methods was positively correlated to NH${}_{4}^{+}$ content. This difference was also positively correlated to the pH and the moisture content but negatively correlated to the nutrient content and VM. This result indicated a loss of culturability in digestates characterized by high pH and moisture content and low nutrient content and VM (characterizing raw digestates and liquid fractions). Indeed, where the pH and moisture content were lowest, i.e., in composted digestates, in one raw digestate and in one liquid fraction, no loss of culturability was observed.

## Discussion

The fate of three pathogenic bacteria found in animal feces was investigated in microcosms containing nine digestates sampled on farms (raw digestates, liquid fractions and composted solid fractions). As indicated by the low level of endogenous *E. coli* (Supplementary Table S3), we hypothesized that the concentration of the three pathogens in the digestates was low. Exogenous cells of each target pathogen were thus inoculated into the digestates.

Different kinds of behavior were observed for the three pathogens. Considering quantification by the culture method and qPCR assay, *L. monocytogenes* survived much longer than *Salmonella* Derby and *C. coli.* Using culture, Nicholson et al. (2005) reported that *L. monocytogenes* in stored slurry and sewage survived longer (up to 6 months) than *E. coli* O157, *Salmonella* and *Campylobacter* (up to 3 months). The higher persistence of *L. monocytogenes* had previously been reported by Hutchison et al. (2005) in studying livestock waste samples. In this paper, *L. monocytogenes* was detected up to 40 days in 6 of the 9 digestates using qPCR assays which is in agreement with the results of Desneux et al. (2016) who using qPCR, detected up to 63 days two strains of *L. monocytogenes* inoculated in pig manures and stored at 20°C. *L. monocytogenes* is known to be particularly resistant to environmental factors (Avery et al., 2014) and to grow over a wide range of temperature and pH conditions (Bille and Doyle, 1991). Its genome contains an extensive repertoire of genes encoding transport proteins and regulators, a characteristic of the genome of ubiquitous bacteria (Vivant et al., 2013). Interestingly, in the present study, culturable *L. monocytogenes* was detected after 40 days only in one digestate (Dig7), highlighting a relatively low persistence of the culturable cells in digestates whereas Desneux et al. (2016) detected a longer persistence of culturable *L. monocytogenes* in manure. This suggests that digestates may represent a less favorable environment than manure for the survival of *L. monocytogenes*. *C. coli* were rapidly reduced in the nine digestates, especially when it was quantified by culture methods. Some studies have shown that *Campylobacter* sp. is a fragile bacterium and appears to be rapidly inactivated in animal by-products (Nicholson et al., 2005; Sinton et al., 2007). But these results were based on enumeration using culture methods, not taking into account a potential VBNC state. In our analysis, the reduction of *C. coli* was lower when it was measured by qPCR than by the culture method (**Figure 2**). This result is in accordance with previous studies that showed good persistence of *Campylobacter* sp. in digested sludge or compost when molecular methods were used (Horan et al., 2004; Wagner et al., 2009; Inglis et al., 2010). McAllister and Topp (2014) reported a long persistence of *C. coli*, above 112 days, in manure samples. Inglis et al. (2010) demonstrated that *C. coli* can survived over 10 months in manure compost. As observed for *L. monocytogenes*, our results seemed to indicate a low persistence of *C. coli* in digestates compared to manure samples. It is noteworthy that the temperature of storage of the microcosms (24°C) could be unfavorable for the persistence of *C. coli* as reported by Bui et al. (2011). These authors investigated the effect of temperature on the survival of *C. coli* in stored swine manure and demonstrated that this bacterium survived only 6 days at 22°C. The persistence of *Salmonella* Derby was intermediate between those of *L. monocytogenes* and *C. coli*.

Several abiotic factors can affect the persistence of pathogenic bacteria. The characteristics of manure samples, including temperature, moisture content and particle size, may contribute to the persistence of pathogens (Ahmed et al., 1995; Garcia et al., 2010; Diao et al., 2015). In this study, the temperature was maintained at 24°C and we focused on such characteristics of the digestates as moisture content, nutrient content, VM and pH. Such physical and chemical characteristics measured in digestates differentiated the three composted or dried digestates (Dig7–9) from the others (raw digestates and liquid fractions). In fact, composted digestates in contrast to raw digestates and liquid fractions were characterized by lower moisture content and higher nutrient contents and VM. The persistence of *Salmonella* Derby was positively correlated with the nutrient content and VM and negatively correlated with the moisture content (**Table 5**), indicating a better persistence of *Salmonella* Derby in composted digestates. Results of Pietronave et al. (2004) confirmed that, under proper storage conditions, finished compost can still provide bio-available nutrients to support *Salmonella* sp. growth. Lemunier et al. (2005) demonstrated the long-term survival of *Salmonella* Enteridis in stabilized compost compared to less mature ones. In contrast to *Salmonella* Derby, we observed a negative correlation between the persistence of *L. monocytogenes* and the pH, nutrient content and VM and a positive correlation with the moisture content (**Table 5**), indicating a lower persistence of *L. monocytogenes* in composted digestates. Klein et al. (2011) had previously reported a higher inactivation rate of *L. monocytogenes* in manure compost than in a manure stockpile. However, a lower persistence was also observed in non-composted digestates (raw digestate Dig3 and liquid fraction Dig6), suggesting that the composting treatment did not alone explain the behavior of *L. monocytogenes*. It is noteworthy that Dig3 and Dig6 were intermediate between the two composted digestates and the other five digestates in terms of nutrient content, pH range and VM. The different behavior between *Salmonella* Derby and *L. monocytogenes* in composts has also been observed by Lemunier et al. (2005). The persistence of seeded *Salmonella* Enteritidis was demonstrated in all composts irrespective of their maturity, whereas the persistence of *L. monocytogenes* was associated to the degree of maturity. Lemunier et al. (2005) explained this result by an increase in the antagonistic effects of the indigenous microflora. Paniel et al. (2010) suggested also that in compost samples, the indigenous microflora appeared to play a significant role in suppression of *L. monocytogenes*, *Salmonella* Infantis and *Enterococcus faecalis*. Some authors have shown that indigenous microflora may limit the persistence of pathogens by limiting availability of nutrients and by indigenous antimicrobial activity (Sidhu et al., 2001; Erickson et al., 2014b). Furthermore, it has been reported that composts contain fungi and *Actinomycetes* which produce substances inhibiting bacterial pathogens (Franke-Whittle et al., 2014). The higher resistance of *Salmonella* to antimicrobial compounds in comparison to *L. monocytogenes* (Patel et al., 2014), may explain that, in our study, the persistence of *L. monocytogenes* was more impacted by the compost than *Salmonella*. A significant correlation with the persistence of *C. coli* was found only with the ammonia (NH${}_{4}^{+}$ /NH_3_) content. NH${}_{4}^{+}$, which was measured in this study, was in equilibrium with NH_3,_ a toxic compound. Depending on the conditions of AD, including temperature and pH, the equilibrium between NH${}_{4}^{+}$ and NH_3_ can be modified. Given the pH values of the nine digestates (ranging between 7.0 and 8.8), the NH_3_ form is well represented (1–26%, data not shown) suggesting that its toxic effect is not negligible. In digestate samples, Orzi et al. (2015) reported a reduction of pathogen concentrations because of NH_3_ production. Interestingly, in our study, NH${}_{4}^{+}$ /NH_3_ content negatively correlated with the persistence of *C. coli* when measured by the culture method, though not by qPCR, suggesting that NH${}_{4}^{+}$ /NH_3_ may have a negative effect only on the culturability of *C. coli* but not on its viability.

Our study clearly demonstrated that composting and NH${}_{4}^{+}$ /NH_3_ content affected the persistence of seeded pathogens. However, the differences between non-composted digestates may be explained by other factors than those measured here. Among such physical and chemical properties, volatile acids were not tested in our study, but this factor has been shown to affect the persistence of *L. monocytogenes* in manure-based compost mixtures of animal origin (Erickson et al., 2014a). Characteristics of environmental matrices can thus contribute to the persistence of pathogens, as suggested by Diao et al. (2015). In particular, the effect on the persistence of pathogenic bacteria of biotic factors such as microbial diversity and activity still remains to be explored in treated waste (Sidhu et al., 2001; Paniel et al., 2010; Klein et al., 2011; Orzi et al., 2015). It is noteworthy that the best persistence of *Salmonella* derby and *L. monocytogenes* estimated by culture was observed for Dig7 which had lowest moisture content (25%), unfavorable to microbial activity (Tate and Terry, 1980).

The persistence of pathogens in environmental matrices (such as soil, manure or compost) has been estimated in the literature mainly by culture methods (Nicholson et al., 2005; Paniel et al., 2010; Locatelli et al., 2013). In environmental conditions, bacteria are submitted to various stresses and lose their culturability, what has been called a Viable but Non-culturable (VBNC) state (Oliver, 2010; Li et al., 2014). To circumvent their loss of culturability, several authors have used DNA-based quantification (qPCR) to study bacteria in environmental samples such as soil, wastewater, sludge, manure or compost (Wéry et al., 2008; Klein et al., 2011; Fu et al., 2015). One of the main criticisms of the use of qPCR for quantifying live bacteria in environmental samples is that it may overestimate the concentration of viable bacteria since extracellular DNA or DNA from non-viable cells can also produce a PCR signal (Nocker and Camper, 2006). However, it was also assumed that extracellular DNA from non-viable cells in manure harboring an abundant and active microflora is quickly degraded and recycled by the microbial populations (Lebuhn et al., 2005; Klein et al., 2011). Furthermore, Fu et al. (2015) demonstrated that the persistence of *Salmonella* sp. and *Shigella* sp. during AD or sludge storage was very similar as indicated by qPCR or reverse transcription qPCR which are both indicators of bacterial cell viability. Desneux et al. (2016) reported that in pig manure or storage lagoons inoculated with *L. monocytogenes*, the quantification of DNA by qPCR gave similar results to those obtained by qPCR combined with propidium monoazide treatment, indicating that the concentration of viable cells was not overestimated by qPCR. In our study, for the three pathogenic species, the decimal reduction was lower in qPCR assays than by the culture method in several digestates, suggesting potential induction of a VBNC state. For all digestates presenting comparatively higher values, whether obtained by qPCR or culture methods, there was at least one of the qPCR signals (i.e., gene copies of *C. coli*, *L. monocytogenes* or *Salmonella* Derby) which decreased very fast (>2 Log in 7 days) confirming that DNA from non-viable cells was rapidly degraded. Additionally, experiments in microcosms inoculated with killed cells of the three pathogens were carried out and the qPCR values were compared with those obtained in microcosms inoculated with live cells. In all but one digestate, the qPCR signal obtained from DNA extracted from microcosms inoculated with killed cells was below those obtained from DNA extracted from microcosms inoculated with live cells. This validated that DNA released by non-viable cells was rapidly degraded. In Dig7, the PCR signals from killed cells was not below those from live cells (**Table 3**), suggesting that DNA from killed cells was not degraded but persisted over time. Dig7 was significantly drier (25% moisture content) than the other digestates (56–97% moisture content). Such low moisture content could thus explain the persistence of DNA observed in the microcosm inoculated with killed cells. In fact, low humidity is known to affect DNAse activity (Blum et al., 1997). At the end, except for Dig7, we assume that the concentrations of gene copies measured by qPCR in the microcosms inoculated with live bacteria represented viable cells. The potential presence of VBNC of *C. coli, Salmonella* Derby and *L. monocytogenes* in matrices such as digestates is not surprising in so far as studies have reported a VBNC state for all the three bacteria in environmental matrices (Oliver, 2010). Using ethidium monoazide and the qPCR technique, Inglis et al. (2010) quantified VBNC of *C. jejuni* in bovine manure compost and Desneux et al. (2016) showed a VBNC state of *L. monocytogenes* in pig manure.

According to some authors, the VBNC state is a survival strategy adopted by many bacteria in response to adverse environmental conditions (Li et al., 2014). Bacteria in a VBNC state lose their culturability on microbial media but retain their cellular integrity and such viability as respiration, enzyme activity and gene expression. The induction of a VBNC state has been already reported in some studies dealing with the treatment of organic waste (Bui et al., 2011; Klein et al., 2011; Leblanc-Maridor et al., 2011; Desneux et al., 2016) and, more specifically, after an AD process (Qi et al., 2008; Viau and Peccia, 2009). Regrowth observed during the storage of digestate, compost or organic waste could be due to a regain of pathogen culturability under favorable conditions after a state of a VBNC induced by an AD process. A recent study by Fu et al. (2015) demonstrated that *Salmonella* sp. and *Shigella* sp. did not actually regrow (no increase of DNA content or cell division) but were reactivated from a VBNC state to a culturable state. Bacteria are capable of regain their culturability under favorable conditions and thus, in the case of pathogenic bacteria pose potential health risks. Hence, culture techniques appear to be ill-suited to evaluate (i) the real efficiency of biological treatment such as AD; and (ii) the biological safety of a digestate intended for spreading on agricultural land. In our experiments, the occurrence of a potential VBNC state was more frequent for *C. coli* (5 of the 9 digestats) and *L. monocytogenes* (4 of the 9 digestates) than for *Salmonella* Derby (**Table 3**). Although VBNC cells of *Salmonella* have been evidenced in manured soil using RT-PCR (Garcia et al., 2010), the majority of digestates (7 of 9) showed no loss of culturability of *Salmonella* Derby. On the other hand, in the case of *C. coli*, 5 of the 9 digestates showed a potential VBNC state and the underestimation by the culture method was of several orders of magnitude after only 7 days. Health risks linked to *Campylobacter* sp. may thus significantly be underestimated when evaluated only with culture-dependent techniques. Interestingly, for *L. monocytogenes*, a potential VBNC state was detected only after 7 days of storage, suggesting that a VBNC state can be acquired only after a relatively long period. Finally, the fact that the pathogenic bacteria did not lose their culturability in all digestates showed that the entry into a VBNC state does not depend only on the species but also on environmental conditions (Desneux et al., 2016). The temperature used (24°C) which is unfavorable to the survival of *C. coli* (Bui et al., 2011) could be a factor of induction of VBNC state and explain why VBNC state was prevalent for this pathogen in our experimental conditions. In our study, we observed that each of the three pathogenic bacteria did not enter into a VBNC state in the same digestates, showing that factors inducing loss of culturability are different between the three species: in the case of *L. monocytogenes*, a potential VBNC state was induced in raw digestates and liquid fractions (except Dig3 and 6) but not in composted digestates. Also, a positive correlation between NH${}_{4}^{+}$ /NH_3_ content and the potential inducing of a VBNC was found in *C. coli* and *L. monocytogenes* microcosms, suggesting that the negative effect of NH${}_{4}^{+}$ /NH_3_ on bacterial culturability could represent a stress leading to a VBNC state. The link between the nature of the digestates and the induction of VBNC state needs further investigation.

## Conclusion

This study has demonstrated that *L. monocytogenes* showed greater persistence in digestates than both *C. coli* and *Salmonella* Derby, probably due to its ubiquitous nature. For three pathogens, a potential VBNC state was induced in digestates, especially for *C. coli* and *L. monocytogenes*. There is a concern for the possibility of regrowth by these two pathogens during the storage of digestate. We have demonstrated the importance of the use of molecular tools such as qPCR for monitoring pathogenic bacteria in environmental samples since culture methods may underestimate bacterial cell concentration. The potential regrowth observed in some final products used for land spreading could be due to a regain to culturability of bacteria fallen into a VBNC state. This occurrence of VBNC cells caused difficulty in evaluating the real efficacy from a sanitary perspective of an AD process and, also, the biological safety of digestates. The persistence of pathogens and the potential induction of a VBNC state were digestate-dependent. We were able to demonstrate: (i) the effect of composting a digestate, which reduced the persistence of seeded *L. monocytogenes* but promoted the maintenance of *Salmonella* Derby; and (ii) the effect of NH${}_{4}^{+}$ /NH_3_ on the culturability of *C. coli* and *Salmonella* Derby. There is a need to investigate other factors, including the influence of biological factors such as the activity and diversity of endogenous microflora and biochemical factors such as the bio-availability of organic matter. In fact, improved knowledge of such parameters may enable us to understand microbial regrowth and/or recontamination as observed in stored digestates in order to improve the management of products from AD processes, thus enhancing their profitable use in agriculture.

## Author Contributions

GM, A-MP, and NW designed laboratory work. GM, CD, and A-MP sampled digestates. GM and CZ performed laboratory work. AC participated in part of the laboratory work and the sampling. GM, A-MP, and NW analyzed data. GM drafted the manuscript. A-MP, NW, and CD finalized the manuscript. All the authors read and approved the final manuscript.

## Conflict of Interest Statement

The authors declare that the research was conducted in the absence of any commercial or financial relationships that could be construed as a potential conflict of interest.
