# Supplementary material for: Persistence and Potential Viable but Non-culturable State of Pathogenic Bacteria during Storage of Digestates from Agricultural Biogas Plants
Source: Front Microbiol. 2016 Sep 14;7:1469. doi: 10.3389/fmicb.2016.01469 (PMC5026136; doi:10.3389/fmicb.2016.01469)
Supplement: Supplementary file 1 [file Presentation_1.PDF]

Supplementary Material

Persistence and potential Viable but Non-Culturable state of pathogenic bacteria during storage of digestates from agricultural biogas plants

G. Maynaud, A.-M. Pourcher, C. Ziebal, A. Cuny, C. Druilhe, J.-P. Steyer and N. Wéry*

*** Correspondence:** Nathalie Wéry, nathalie.wery@supagro.inra.fr

A.

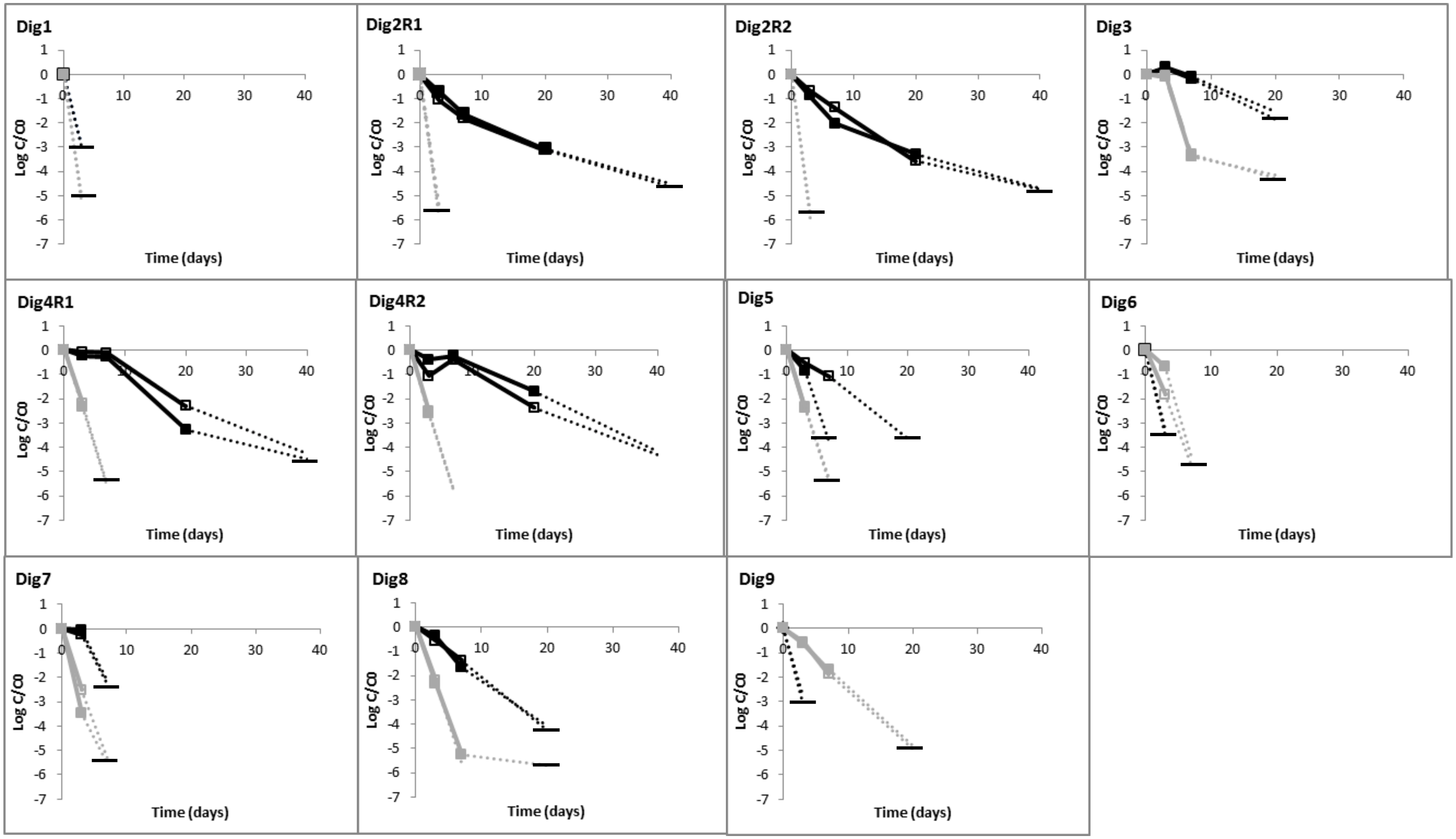
Dig1
Dig2R1
Dig2R2
Dig3
Dig4R1
Dig4R2
Dig5
Dig6
Dig7
Dig8
Dig9
Log C/C0
Time (days)

B.

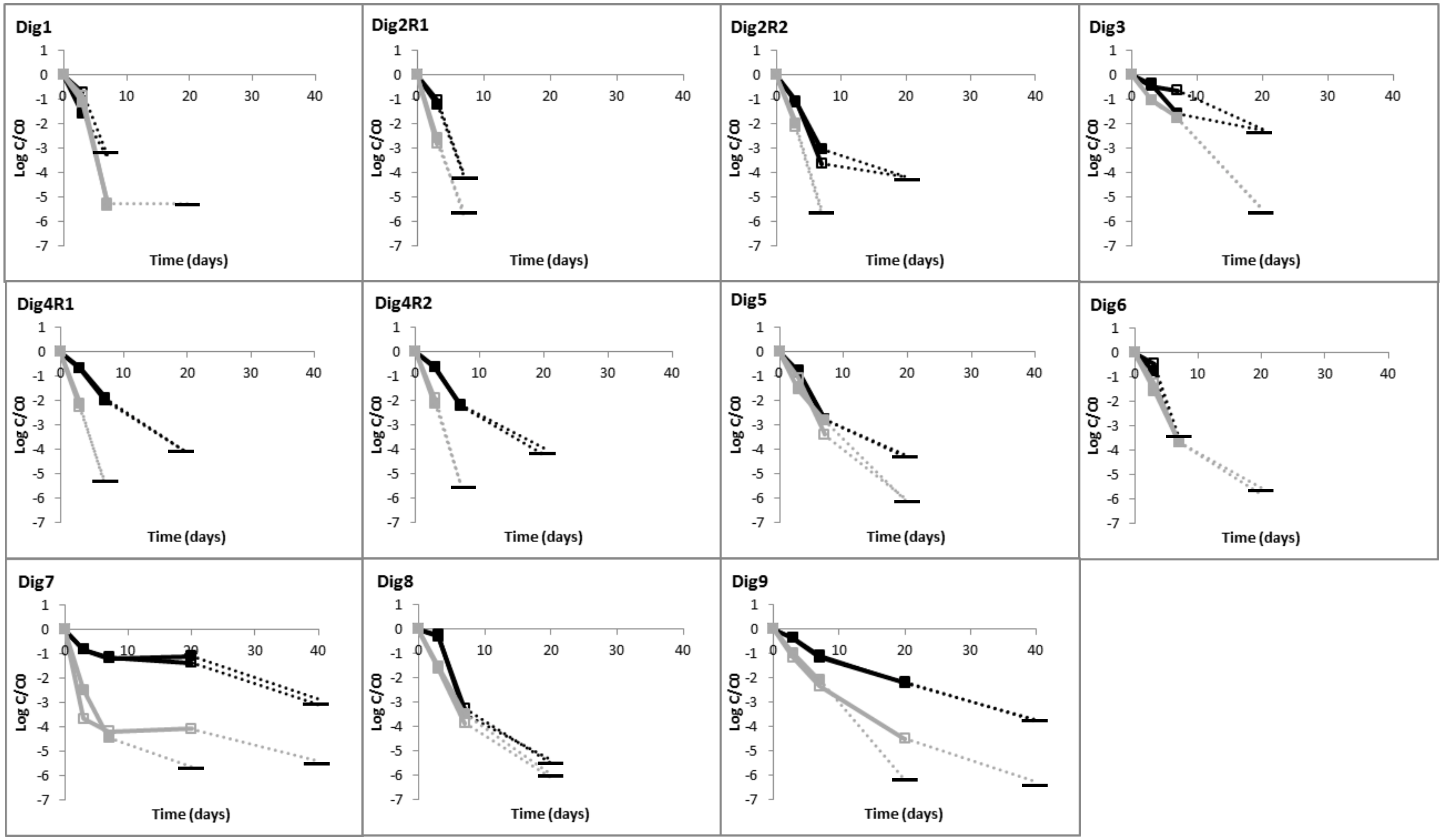
Dig1
Dig2R1
Dig2R2
Dig3
Dig4R1
Dig4R2
Dig5
Dig6
Dig7
Dig8
Dig9
Log C/C0
Time (days)
1
0
-1
-2
-3
-4
-5
-6
-7
0
10
20
30
40

C.

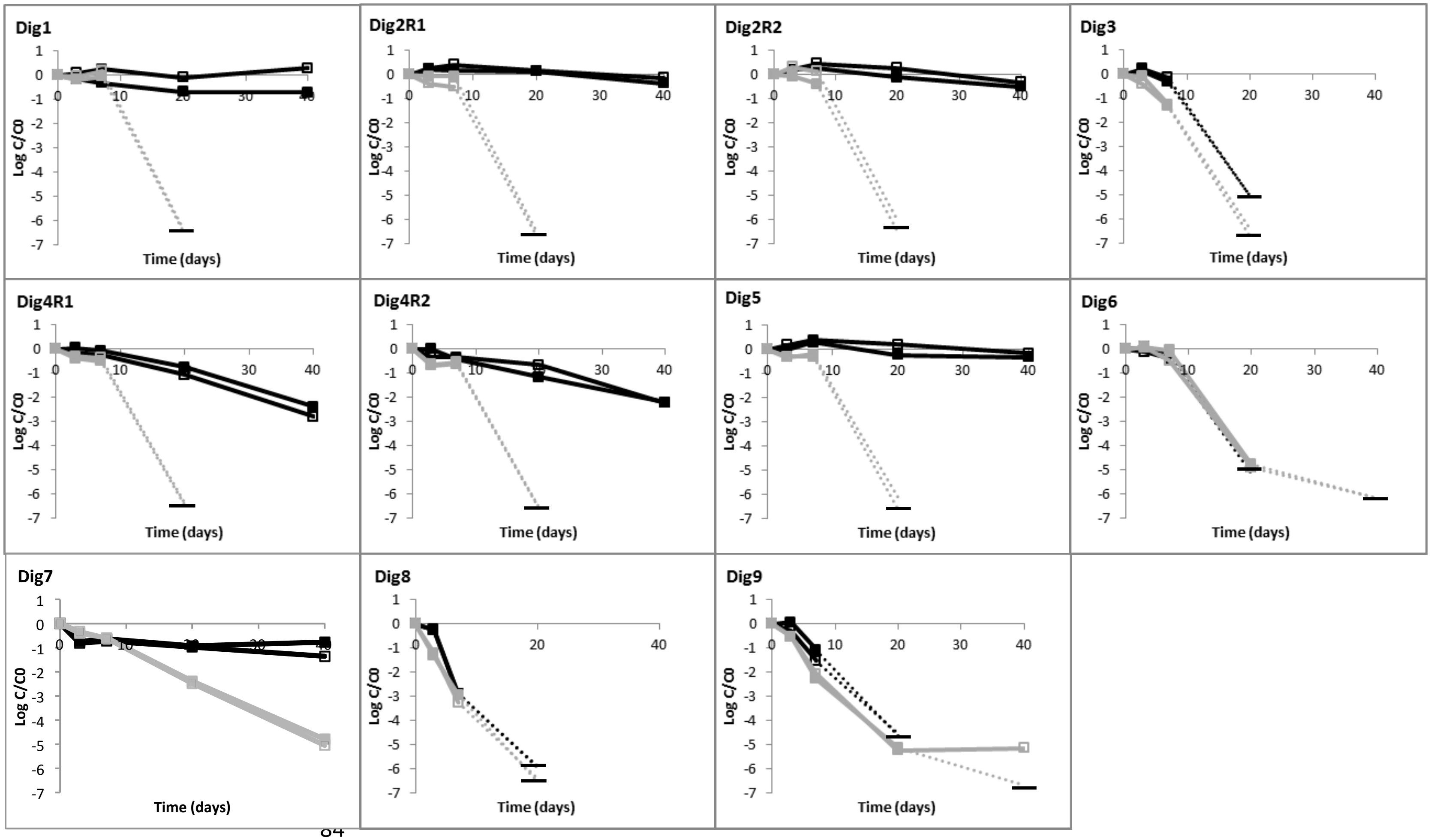

Figure S1. Persistence of *C. coli* (A), *Salmonella* Derby (B) and *L. monocytogenes* (C) measured by culture method (grey curve) and qPCR assay (black curve) in duplicates for each digestate. Horizontal Black line represented the detection limits for each sample. The last point connected by a dotted line represented the first point below the detection limit.

Supplementary Table S1. Mean, standard deviation (SD) and **c**oefficient of variation (CV %) of Dig2 and Dig4 calculated across duplicate samples (R1 and R2) and for each performed analysis.

Analysis		Dig4			Dig2		
		Mean	SD	CV %	Mean	SD	CV %
pH		8.3	0.0	0.0	8.0	0.0	0.0
Moisture content (%)		93.8	0.0	0.0	91.2	0.0	0.0
VM (%)		3.4	0.1	3.4	3.5	0.0	1.3
TKN (gN kg^{-1})		10.3	0.1	1.3	4.0	0.3	7.7
TOC (gC kg^{-1})		19.5	1.0	5.0	20.5	1.1	5.4
NH_4^+ (g kg^{-1})		8.1	0.2	3.0	3.1	0.1	3.3
K_2O (g kg^{-1})		3.0	0.1	3.8	8.0	0.0	0.0
P_2O_5 (g kg^{-1})		6.1	0.3	5.5	3.4	0.2	4.5
Campylobacter coli	qPCR[1]	7.4	0.1	1.8	6.3	0.3	5.0
	Culture[2]	1.4	0.0	0.0	1.4	0.0	0.0
Salmonella Derby	qPCR	6.0	0.2	2.8	4.4	0.5	11.9
	culture	1.4	0.0	0.0	1.4	0.0	0.0
Listeria monocytogenes	qPCR	8.5	0.2	1.8	9.0	0.2	1.7
	culture	7.3	0.0	0.0	7.3	0.0	0.0

[1]Log of gene copy g^{-1} and [2]Log of CFU g^{-1}, both measured at 7 days.

VM: volatile matter, TKN: total Kjeldalh nitrogen, TOC: total organic carbon.

Supplementary Table S2. Inactivation rate (IR expressed in day^{-1}) and decimal reduction at 7 days (DR expressed in Log) measured by qPCR and culture methods. Only values of one duplicate are presented.

Digestate	*Salmonella* Derby				*Campylobacter coli*				*Listeria monocytogenes*			
	qPCR		Culture		qPCR		Culture		qPCR		Culture	
	IR	DR	IR	DR	IR	DR	IR	DR	IR	DR	IR	DR
Dig1	0.5	3.3	0.8	5.2	1.0	2.9	1.7	5.0	0.0	0.3	0.5	0.0
Dig2	0.6	4.1	0.9	5.8	0.1	1.8	1.9	5.6	0.0	-0.1	0.5	0.1
Dig3	0.2	1.6	0.3	1.7	0.1	0.2	0.8	3.3	0.4	0.3	0.3	1.2
Dig4	0.3	1.9	0.7	5.5	0.2	0.3	0.7	5.4	0.1	0.2	0.5	0.5
Dig5	0.4	2.8	0.5	3.4	0.2	1.1	0.8	5.4	0.0	-0.3	0.5	0.2
Dig6	0.5	3.6	0.5	3.7	1.2	3.5	0.6	4.4	0.4	0.4	0.4	0.1
Dig7	0.1	1.2	0.6	4.2	0.3	2.3	1.2	5.6	0.0	0.7	0.1	0.6
Dig8	0.5	3.5	0.5	3.5	0.2	1.4	0.7	5.6	0.4	2.9	0.4	2.9
Dig9	0.1	1.2	0.3	2.1	1.0	3.1	0.3	1.9	0.2	1.1	0.3	2.3

Supplementary Table S3. Concentration of endogenous *Escherichia coli* measured by culture method in digestates. The mean of three replicates and the standard deviation are given.

Digestate	Concentration of *E. coli* (CFU g^{-1} or mL^{-1})
Dig1	<DL*
Dig2	<DL
Dig3	$4.5\ 10^{1} \pm 1.1\ 10^{1}$
Dig4	<DL
Dig5	$6.4\ 10^{3} \pm 2.1\ 10^{2}$
Dig6	$2.0\ 10^{2} \pm 3.9\ 10^{1}$
Dig7	<DL
Dig8	$4.8\ 10^{3} \pm 1.7\ 10^{3}$
Dig9	<DL

*DL: Detection limit (10 CFU g^{-1} or mL^{-1})
